# Readmissions at a Public Safety Net Hospital

**DOI:** 10.1371/journal.pone.0091244

**Published:** 2014-03-11

**Authors:** Eri Shimizu, Kathleen Glaspy, Mallory D. Witt, Kimble Poon, Susan Black, Shelley Schwartz, Tasneem Bholat, Norma Diaz, Allen Kuo, Brad Spellberg

**Affiliations:** 1 Division of General Internal Medicine, Los Angeles Biomedical Research Institute at Harbor-University of California at Los Angeles (UCLA) Medical Center, Torrance, California, United States of America; 2 Department of Medicine, Hawaii Permanente Medical Group, Wailuku, Hawaii, United States of America; 3 Department of Medicine, Maui Memorial Medical Center, Wailuku, Hawaii, United States of America; 4 David Geffen School of Medicine at University of California at Los Angeles (UCLA), Los Angeles, California, United States of America; 5 Division of HIV Medicine, Los Angeles Biomedical Research Institute at Harbor-University of California at Los Angeles (UCLA) Medical Center, Torrance, California, United States of America; 6 Department of Quality, Harbor-University of California at Los Angeles (UCLA) Medical Center, Torrance, California, United States of America; University of California, San Francisco, United States of America

## Abstract

**Objective:**

We aimed to determine factors related to avoidability of 30-day readmissions at our public, safety net hospital in the United States (US).

**Methods:**

We prospectively reviewed medical records of adult internal medicine patients with scheduled and unscheduled 30-day readmissions. We also interviewed patients if they were available. An independent panel used pre-specified, objective criteria to adjudicate potential avoidability.

**Results:**

Of 153 readmissions evaluated, 68% were unscheduled. Among these, 67% were unavoidable, primarily due to disease progression and development of new diagnoses. Scheduled readmissions accounted for 32% of readmissions and most (69%) were clinically appropriate and unavoidable. The scheduled but avoidable readmissions (31%) were attributed largely to limited resources in our healthcare system.

**Conclusions:**

Most readmissions at our public, safety net hospital were unavoidable, even among our unscheduled readmissions. Surprisingly, one-third of our overall readmissions were scheduled, the majority reflecting appropriate management strategies designed to reduce unnecessary hospital days. The scheduled but avoidable readmissions were due to constrained access to non-emergent, expensive procedures that are typically not reimbursed given our system’s payor mix, a problem which likely plague other safety net systems. These findings suggest that readmissions do not necessarily reflect inadequate medical care, may reflect resource constraints that are unlikely to be addressable in systems caring for a large burden of uninsured patients, and merit individualized review.

## Introduction

Hospital readmissions have been linked to adverse patient outcomes, resulting from potentially modifiable causes, including poor discharge planning and substandard care [1], [2], [3], [4], [5], [6]. However, higher readmission rates have also been related to poverty [7], ethnicity [8], [9], [10], limited education [11], low health literacy [12], Medicaid insurance [13], [14], and poor social supports [15], [16]. It is notable that many of these factors are not under the control of a given hospital, and are unevenly distributed among hospital systems, with public safety net hospitals caring for a larger proportion of these indigent or otherwise at-risk patients. It is not surprising, then, that public hospitals have been associated with higher readmission rates [17], [18]. Nonetheless, readmission rates are used as a measure of quality, irrespective of the patient population served.

Readmissions at public safety net hospitals are not well described. Therefore, we sought to define the frequency and factors associated with avoidable and unavoidable readmissions at our public safety net hospital. We sought to determine whether our readmission rates truly reflect poor quality.

## Methods

### Setting and Patients

This was a prospective, observational, cohort study conducted at Harbor-UCLA Medical Center (HUMC), a tertiary care, level 1 trauma center in Los Angeles County. HUMC is a public safety-net, teaching hospital. The study was determined to be Category 4 Exempt by the John F. Wolf IRB at the Los Angeles Biomedical Research Institute at HUMC.

Medical records were reviewed for adult patients (≥ 18 years old) who were admitted to an internal medicine service and readmitted within 30 days to HUMC on any service, for any reason, between January and September 2012, inclusive of both scheduled and unscheduled readmissions. Readmitted patients were identified by a daily report which was already in place to monitor readmission rates.

### Data Collection

Data was compiled from 1) index admission and readmission charts; 2) available outpatient notes during the interval between admissions; 3) follow-up appointment schedules. Patient demographic data, discharge medications, length of stay, time to readmission, and the primary index and readmission diagnoses were recorded. Major co-morbid conditions were recorded and the Charlson comorbidity index (CCI) score was calculated [19].

Medication reconciliation was performed (ES and KG). A manual comparison was made between medications documented on admission, received in hospital, and documented on discharge. Reconciliation was determined to be complete if all appropriate medications were resumed or discontinued on discharge. Partial completion was considered if 1–2 medications were not reconciled, but were not considered pertinent to the admission or major co-morbid diagnoses (e.g. albuterol as needed for diabetic with intermittent asthma admitted for cellulitis). Incomplete reconciliation was determined if critical medications were not reconciled (e.g. long acting inhalers in patient on chronic therapy for COPD; lactulose for hepatic encephalopathy).

### Interviews

Patient interviews were conducted for patients who remained hospitalized at the time they were identified by the readmission query (ES, KG, and ND). Two attempts were made to carry out interviews. Interpreters were used as necessary. Information gathered from interviews included self-identified ethnicity, primary language, living conditions, perceived medical understanding and barriers to care. Health literacy was evaluated by highest achieved educational level and a three-question screening survey [20].

### Adjudication of Avoidability of Admission

An in-depth chart review was conducted by a reviewing physician (ES or KG), who presented data to a monthly group panel of three internal medicine specialists who were not involved in data abstraction. The panel used a pre-specified set of criteria to adjudicate whether the admission was avoidable or not.

Adapted from Goldfield and colleagues [21], unscheduled readmissions were designated as avoidable if the readmission was for:

1. An ongoing or recurrent condition which led to the initial admission, or for a closely related condition, which could have been prevented by an intervention during the index admission or during a follow-up appointment.
2. An acute decompensation of a chronic medical condition that was not the reason for the initial admission but was plausibly related to care either during or immediately after the initial admission.
3. An acute medical complication plausibly related to care during the initial admission (e.g., patient with a urinary catheter placed in hospital develops a urinary tract infection 10 days later).
4. A surgical procedure to address a continuation or recurrence of the problem causing the initial admission (e.g., a patient readmitted for an appendectomy following an initial admission for abdominal pain and fever).

Intentionally scheduled readmissions were for diagnostic or therapeutic procedures and were designated as avoidable when:

1. A procedure would have been more appropriately completed prior to discharge from the index admission (e.g., surgical removal of indwelling hardware in a patient with septic joint).
2. A procedure was not performed during the index admission but for which there was adequate time to complete (e.g., patient hospitalized for a week with suspected upper GI bleeding, waiting for endoscopy).
3. A procedure could have been performed safely without requiring hospitalization (e.g., endoscopy, colonoscopy, MRI).
4. A procedure was not completed due to absent or inadequate pre-procedural evaluation or clinical indication (e.g. patient readmitted for procedure that specialty service deemed not clinically indicated; specialty consult not obtained prior to readmission).
5. A procedure could not be completed over a weekend during the index admission, but there was reasonable expectation of weekend availability of a treatment or procedure (e.g., chemotherapy administration).

Where there was disagreement among the panel members during initial evaluations, subsequent discussion was undertaken to attempt to resolve the disagreement. Unanimity among the panel was achieved for 143 out of 153 cases. In the 10 cases for which subsequent discussion could not resolve the disagreement, the final adjudication was based on concordance of 2 of the 3 panel reviewers.

Finally, factors that may have contributed to the readmission were identified for each case; more than one allowed. Adapted from Oddone et al and Hughes, these factors were categorized as follows [22], [23]:

1. Disease progression
2. New diagnosis, which could not have been predicted at time of index discharge
3. Patient-specific: medication/appointment/other cares non-adherence, patient choice, alcohol or drug abuse, psychiatric disorder
4. Social: lack of financial resources or housing, inadequate home support, chaotic living situation
5. Clinical: Medication errors, adverse drug reactions, complication of procedures, inappropriate discharge, lab or test error, missed diagnosis, inappropriate medical care
6. Systems-based: Medications not provided, follow-up appointments not made, inadequate teaching, lapse of communication between inpatient and outpatient, appropriate referrals not made (e.g. hospice, home health), unavailable diagnostic or therapeutic modalities.

### Statistical Analysis

We anticipated that one quarter of all readmissions would be avoidable,[24] and that at least 10 readmissions per category (clinical, patient-specific, social, and system-based) would be required to adequately understand the factors that drive readmissions. Thus, we planned to study 120 uniquely readmitted patients (10 patients * 4 categories of avoidable readmissions * 3 [avoidable anticipated to be 1/3 of all readmissions]  =  120 patients) but anticipated that 25% of the readmissions would be patients with more than 1 readmission during the study period. Thus, we planned to study 150 total patients, affording an 80% power to detect a 50% relative reduction in the proportion of patients with individual risk factors present between patients whose readmissions were avoidable or not (α  =  0.05, Chi Squared). Differences between avoidable and non-avoidable admissions were compared by Chi Squared or Fisher’s Exact Test for proportions, as appropriate, and the Mann Whitney U test for continuous variables.

## Results

### Readmitted Population Characteristics

Of 1026 total admissions evaluated, there were 153 30-day readmissions, for a readmission rate of 14.9%. One hundred twenty-nine (84%) of 153 readmissions were readmitted with a diagnosis that was the same or related to the index diagnosis. Of the 30-day readmissions, 104 (68%) were unscheduled and 49 (32%) were scheduled. Of the unscheduled readmissions, 82 (79%) were readmitted with the same or a related diagnosis. One hundred three (67%) of 153 readmissions were unavoidable.

There were 137 unique, readmitted patients, of whom 124 (91%) were readmitted once, 10 (7%) were readmitted twice, and 3 (2%) were readmitted three times. The average CCI was 3.53 and only 23% had a CCI score of zero. The majority of patients were receiving federal, state, or local public healthcare assistance (50%) or were uninsured, self-pay patients (36%) (Table 1). Only nineteen (12%) were Medicare patients and 2% were privately insured. By comparison, the number of inpatient days by payer for all patients at HUMC (including surgical, obstetrical, pediatric) during the study period was as follows: Medi-cal 42.8%, self-pay 17.2%, other government forms of assistance 15.9%, self-insured 14.8%, and Medicare 9.0%.

**Table 1:** Characteristics of All Readmitted Patients.

Demographics	Mean (range) age (yrs)	53.3 (21–82)
	Age 75 yrs or older	10 (7%)
	Males	59%
Insurance	Medicaid	54 (39%)
	Self Pay	49 (36%)
	Medicare	17 (12%)
	Other government programs	14 (10%)
	Private	3 (2%)
Comorbidities	Mean CCI (range)*	3.53 (0–11)
	Hypertension	75 (57%)
	Diabetes mellitus	53 (39%)
	Malnutrition	29 (21%)
	Heart Failure	26 (19%)
	Coronary artery disease	24 (18%)
	Metastatic cancer	23 (17%)
	Substance Abuse	20 (15%)
	All other cancer	18 (13%)
	Psychiatric	17 (12%)
	Obesity	16 (12%)
	Renal failure	26 (19%)
	Lymphoma/leukemia	15 (11%)
	Stroke	13 (10%)
	Liver disease	14 (10%)
	HIV/AIDS	3 (2%)

*CCI  =  Charlson comorbidity index score.

Interviews were conducted for 72 of 137 (53%) patients. The patients who were not interviewed were unavailable at the time the interviews were attempted due to tests or procedures, had been discharged already, or declined the interview. Of those interviewed, 88% were ethnic minorities, with Hispanics comprising 54% of the cohort (Table 2). This was not statistically different from our inpatient general population (54.9% Latino, 23.7% African American, 12.3% non-Hispanic White, and 7.9% Asian). English was the primary language in half of the cohort and Spanish was the most common primary language after English (42%). In addition, our cohort had low educational attainment levels: one third (33%) did not complete the eighth grade of school and another 17% did not complete high school. Consistent with this observation, almost half (43%) reported needing at least some help with reading written material, 86% needing help filling out medical forms, and 54% reported having problems understanding their medical condition from written materials (Table 2).

**Table 2:** Socioeconomic Data from Interviewed Patients (N = 72).

Ethnicity	Hispanic	39 (54%)
	African American	15 (21%)
	Aian/Pacific Islander	9 (13%)
	Non-Hispanic Whlte	7 (10%)
	Other	2 (3%)
Language	English	36 (50%)
	Spanish	30 (42%)
	Other	6 (8%)
Education	Less than 3rd grade	12 (17%)
	4^th^ to 8th grade	12 (17%)
	Some high school	12 (17%)
	Completed high school	13 (18%)
	More than high school	23 (32%)
Needs help reading	Never	40 (56%)
	Sometimes	17 (24%)
	Always	14 (19%)
Needs help to complete forms	Never	10 (14%)
	Sometimes	30 (42%)
	Always	32 (44%)
Problems understanding*	Never	32 (44%)
	Sometimes	27 (38%)
	Always	12 (17%)

*Problems understanding their medical condition from written materials.

### Unscheduled Readmissions

Of the 104 unscheduled readmissions, 69 (66%) cases were designated as unavoidable (Table 3). Demographic and clinical factors were generally similar between avoidable and unavoidable readmissions (Table 3 and 4). However, readmissions determined avoidable were less likely to have attended the follow-up appointment. The most common reasons for unavoidable readmissions were malignancy complications, heart failure, renal failure, respiratory failure, and chemotherapy. The most common reasons for avoidable readmissions were malignancy complications, heart failure, cellulitis, and other infection.

**Table 3:** Characteristics of Patients with Unavoidable vs. Avoidable, Unscheduled Readmissions.

		Unavoidable (N = 69)	Avoidable (N = 35)	p value
Mean Age		52	55	0.3
Male Gender		61%	57%	0.9
Insurance	Self Pay	36%	46%	0.8
	Medicaid	36%	37%	
	Medicare	15%	9%	
	Private	3%	3%	
	Other	10%	6%	
Mean CCI*		3.51	3.34	0.6
Mean Index LOS† (d)		6	5	0.9
Time to readmission	≤ 1 week	35%	34%	0.9
	1–2 weeks	22%	20%	
	2–3 weeks	22%	23%	
	>3 weeks	22%	23%	
Mean # Discharge Meds		4	4	0.9
Med Reconciliation done?	No	4%	11%	0.3
	Yes	78%	69%	
	Partial	17%	17%	
	Unsure	0%	3%	
Mean Time to Follow-up (d)		10	14	0.4
Follow-up Time Interval	≤ 1 week	46.4%	42.9%	0.7
	1–2 weeks	29.0%	20.0%	
	2–4 weeks	11.6%	14.3%	
	> 4 weeks	8.7%	14.3%	
	N/A	4.3%	8.6%	
Discharge Summary Dictated	≤ 1 week	78%	60%	0.09
	≤2 week	7%	20%	
	≤4 week	9%	6%	
	>1 week	6%	14%	
		Unavoidable (N = 57)	Avoidable (N = 30)	
Attended follow-up at HUMC (N = 87)	No	3 (5%)	7 (23%)	0.02
	Yes	42 (74%)	13 (44%)	
	Future Appt	11 (19%)	8 (27%)	
	Unsure	1 (2%)	2 (7%)	

*CCI  =  Charlson comorbidity index score.

† LOS  =  Length of Stay.

**Table 4:** Diagnoses of Patients with Unscheduled Unavoidable vs. Avoidable Readmissions.

Diagnosis	Unavoidable (N = 69)	Avoidable (N = 35)	p value
Malignancy complication	9 (13%)	4 (11%)	0.5
Heart Failure	6 (9%)	4 (11%)	0.8
Cellulitis	5 (7%)	3 (9%)	0.7
Other Infections	5 (7%)	4 (11%)	0.9
Renal Failure	6 (9%)	2 (6%)	0.5
Respiratory Failure	7 (10%)	1 (3%)	0.2
Malignancy workup	5 (7%)	2 (6%)	0.6
NSTEMI/Unstable Angina	5 (7%)	2 (6%)	0.6
Decompensated Cirrhosis	5 (7%)	2 (6%)	0.6
Chemotherapy	6 (9%)	0	0.1
Diabetic Gastroparesis	5 (7%)	1 (3%)	0.4
Sepsis	2 (3%)	2 (6%)	1
Atrial Fibrillation	2 (3%)	2 (6%)	1
Drug overdose or withdrawal	3 (4%)	1 (3%)	0.7
Pneumonia	3 (4%)	0	0.4
Delirium	3 (4%)	0	0.4
Electrolyte abnormalities	1 (1%)	2 (6%)	0.3
UTI/Pyelonephritis	0	2 (6%)	0.1
Dehydration	2 (3%)	0	0.7
Adverse Drug Reaction	2 (3%)	0	0.7
Fracture	1 (1%)	0	1

Data for follow-up appointment attendance were available for 87 of the 104 (84%) patients with unscheduled readmissions (Table 3). Fifteen patients were followed outside our facility and 2 left against medical advice. As a group, 63% saw a physician prior to readmission, but only 44% of avoidable readmissions attended the follow-up appointment versus 74% of unavoidable readmissions (p  =  0.02). Although not statistically significant, avoidable readmissions also tended to be associated with a longer time interval between discharge and follow-up appointment; less likely to have medications appropriately reconciled; and to not have had a timely discharge summary dictated.

Among the unavoidable readmissions, 57% of cases were attributed to disease progression, 29% attributed to new diagnoses, and 20% had at least one patient specific factor contributing to readmissions (Table 5). In contrast, among avoidable readmissions, only 6% were attributed to disease progression and none attributed to new diagnoses. However, 62.9% of cases were attributed to at least one clinical factor (e.g. medication error, adverse drug reaction, missed diagnosis, complication of diagnostic procedure) and 48.6% attributed to system-based factors (e.g. follow up appointments not made, inadequate teaching, referrals to home health not made, unavailable diagnostic or therapeutic capabilities) (Table 5). Patient factors (e.g. medication noncompliance, appointment noncompliance, underlying substance abuse, psychiatric disorder, etc.) were attributed to 16 (18.8%) of unscheduled, unavoidable readmissions.

**Table 5:** Factors Associated with Unavoidable vs. Avoidable, Unscheduled Readmissions.

	Unavoidable (N = 69)	Avoidable (N = 35)	P value
Disease Progression	39 (57%)	2 (6%)	< 0.001
New Diagnosis	20 (29%)	0	< 0.001
Healthcare System Resource Factors	2 (3%)	17 (49%)	< 0.001
Suboptimal Clinical Care	5 (7%)	22 (63%)	< 0.001
Patient Factors	14 (20%)	5 (14%)	0.32
Social Factors	2 (3%)	2 (6%)	0.96

### Scheduled Readmissions

All 49 scheduled readmissions were re-hospitalized for interventions relating to an underlying condition present on index admission. The most common diagnosis was an established or suspected malignancy (69% cases). Among scheduled readmissions, the 5 most common reasons for readmission were chemotherapy for malignancy (18, 37%), malignancy workup (15, 31%), renal failure (4, 8%), heart failure (2, 4%), and sepsis (2, 4%).

The majority of cases (69%) were designated as unavoidable due to patients being admitted for procedures that were: 1) an appropriate next step to workup or treatment (e.g., invasive biopsy, chemotherapy); or 2) unable to be performed on index admission (e.g. CT guided biopsy over a weekend) (Table 6). However, almost a third of scheduled readmissions were designated as avoidable (Table 6). The most common cases cited as avoidable were for: 1) procedures that were unavailable over a weekend, and for which there was reasonable expectation that it would be available at other institutions (e.g. endoscopy or colonoscopy); or 2) procedures that could have been completed either as an outpatient or during the index admission.

**Table 6:** Unavoidable vs. Avoidable Scheduled Readmissions.

Readmission Reason		Unavoidable	Avoidable
Chemotherapy		20	1
Surgery		7	4
Procedures	EGD/Colonoscopy	0	6
	ERCP	1	0
	Renal Biopsy	2	1
	Expedited Cancer Work-up	1	1
	CT guided biopsy	3	1
	BM Biopsy	0	1

## Discussion

In our analysis of readmissions at a public, safety net hospital in the US we determined that only one third of readmissions were potentially avoidable. Importantly, our evaluations were based on clinical rather than administrative data with results that are concordant with previous studies that used a similar methodology [24], and are much lower than the estimated 76% avoidability reported by the US Medicare Payment Advisory Committee, which used administrative data in their assessment [25]. Administrative analyses designate any readmission with the same or a related diagnosis as potentially avoidable, an oversimplification that does not reflect the reality that diseases severe enough to require initial hospitalization often progress despite treatment. Indeed, had we applied an administrative-level definition for avoidability of readmissions, 84% of our entire cohort and 79% of the unscheduled cohort would have been classified as avoidable because they were readmitted with the same or a related diagnosis.

Additionally, our study demonstrates the important role of scheduled readmissions—they are common and usually clinically indicated. Examples of necessary and legitimate readmissions we encountered included the following: repeat cycles of chemotherapy and follow up surgical procedures that were delayed to enable medical optimization prior to surgery. In our study, fully one third of our readmissions were scheduled, three times the 10% estimate by Jencks et al [26], and most of these were deemed appropriate by the independent review panel. Appropriate scheduled readmissions are used to decrease cost and improve patient safety, by reducing hospital stays.

While a third of these scheduled readmissions were considered avoidable, these readmissions were largely due to resource limitations inherent in a public, safety net hospital. For example, non-emergent, expensive procedures (such as endoscopy or MRI), which typically are not reimbursed given our payor mix, were limited in availability over weekends or holidays. We considered these readmissions avoidable in the context of what would reasonably be available at other institutions, where external payors bear the cost burden of paying for the procedures, and the physician billing associated with them, because the patients cared for are insured. Although it may seem reasonable to expect the availability of these services, such expectations of a safety net system with limited resources may not actually be possible. Interventions to reduce avoidable readmissions of this nature—such as employing endoscopy staff on weekends and holidays, or extending weekday hours—would be expensive. Particularly in an era of constrained government budgets, such system changes are problematic for public, safety net hospitals, which care primarily for unfunded patients and do not receive reimbursement for most procedures. The increased cost would require increased taxpayer dollar input and would not be offset by increased revenues, in contrast to private hospitals.

In our analysis of unscheduled readmissions, cancer complications (12.5%), heart failure (9.6%), renal failure (7.7%), respiratory failure (7.7%), and cellulitis (7.7%) were the top five diagnoses. This variability in case mix has an important implication in preventing readmissions as heart failure is likely to require a very different strategic intervention than preventing readmissions in cancer patients. Cancer readmissions are also more likely to be scheduled and appropriate. In our cohort, 17 (77%) of cancer patients had scheduled readmissions within 30 days. Additionally, complications due to cancer, including chemotherapy side effects (e.g. neutropenic fever), mass effects (e.g. bowel obstruction), or overall increased susceptibility to infections due to high healthcare exposures or suppressed immunity are harder to anticipate and prevent.

In this cohort, avoidable, unscheduled readmissions were associated with inappropriate care or discharge (31.4%), delayed follow-up appointments (20%), and incomplete medication reconciliation (14.3%). Interventions to mitigate some of these factors— e.g., scheduling earlier follow-up appointments and ensuring medication reconciliation at admission and discharge—would require minimal cost and potentially decrease readmission rates in our US public, safety net hospital. Several studies have shown that utilizing discharge planners, post-discharge nursing and pharmacy phone calls to reconcile medications, and improved communication to outpatient care providers decrease thirty day readmission rates [27], [28], [29]. These strategies merit study in the future in our patient population.

Furthermore, like other public safety-net hospitals, we provide care predominantly to the uninsured and underinsured, poorly educated patients, and patients with a high co-morbidity index, all of which have been associated with higher readmission rates [9], [17], [30]. Several studies have reported health literacy and patient comprehension of medical care to be important factors in transition of care at discharge [31], [32], [33]. Our study supports this premise as interviewed patients in this cohort reported having problems understanding their conditions (54%) and needing help reading written materials (43%). Addressing issues of poverty and education would require a comprehensive intervention and significant investment of already scarce resources.

A primary limitation of our study was its single-center nature, focusing on readmissions at an urban, public hospital. These findings cannot necessarily be extrapolated to broader healthcare systems, and to private hospitals in particular. Another limitation was the potentially subjective nature by which avoidable admissions were judged. However, the only alternative was to use administrative definitions, which, as noted above, are likely to be substantially less accurate because they ignore fundamentally important clinical factors. We attempted to limit the impact of subjectivity by using pre-specified, objective criteria to define avoidable admissions and using an independent adjudication panel comprised of internal medicine faculty who were not involved in data abstraction. The fact that unanimity was achieved among the panel in the vast majority (90%) of cases provides reassurance that a level of objectivity was achieved.

In conclusion, our analysis of readmissions at our public safety net hospital challenges the notion that readmissions necessarily reflect poor quality. Scheduled readmissions, which accounted for a third of all readmissions in this study, served to reduce hospital days and actually improved quality. Readmissions are complex phenomena, resulting at times from lapses in quality, but also from immutable factors such as inexorable disease progression, development of new diagnoses in medically complex patients, system-based factors, as well as economic, educational, and demographic factors.
